# Comprehensive molecular characterization of inhibitors of apoptosis proteins (IAPs) for therapeutic targeting in cancer

**DOI:** 10.1186/s12920-020-0661-x

**Published:** 2020-01-21

**Authors:** Jianfeng Liang, Wanni Zhao, Pan Tong, Ping Li, Yuanli Zhao, Hua Li, Jun Liang

**Affiliations:** 1 Department of Neurosurgery, Peking University International Hospital, 1 Science Park Road, ZGC Life Science Park, Beijing, 102206 China; 2 General Surgery Department, Beijing Hospital, National Center of Gerontology; Institute of Geriatric Medicine, Chinese Academy of Medical Sciences, No.1 DaHua Road, Dong Dan, Beijing, 100730 China; 3 Department of Bioinformatics and Computational Biology, The University of Texas M. D. Anderson Cancer Center, Houston, TX 77030 USA; 4 Department of Hematology, Tongji Hospital of Tongji University, 389 Xincun Road, Shanghai, 200065 China; 5 Department of Neurosurgery, Beijing Tiantan Hospital, Capital Medical University, 6 Tiantan Xili, Beijing, 100050 China; 6 State Key laboratory for Oncogenes and Bio-ID Center, School of Biomedical Engineering, Shanghai Jiao Tong University, 800 Dongchuan Road, Shanghai, 200240 China; 7 Department of Oncology, Peking University International Hospital, 1 Science Park Road, ZGC Life Science Park, Beijing, 102206 China

**Keywords:** Drug sensitivity, Gene expression regulation, Inhibitors of apoptosis proteins, Personalized cancer therapy, Prognostic factors

## Abstract

**Background:**

Inhibitors of apoptosis proteins (IAPs) are a family of antiapoptotic proteins modulating cell cycle, signal transduction and apoptosis. Dysregulated IAPs have been reported to contribute to tumor progression and chemoresistance in various cancers. However, existing studies were sporadic and only focus on one specific cancer with one particular gene in the IAPs family. A systematic investigation on the co-expression pattern, regulation frameworks on various pathways, prognostic utility on patient outcomes, and predictive value on drug sensitivity among all the IAPs across multiple tumor types was lacking.

**Methods:**

Leveraging The Cancer Genome Atlas data with comprehensive genomic characterizations on 9714 patients across 32 tumor types and the Genomics of Drug Sensitivity in Cancer data with both genomic characterizations and drug sensitivity data on > 1000 cell lines, we investigated the co-expression pattern of IAPs, their regulations of apoptosis as well as other pathways and clinical relevance of IAPs for therapeutics development.

**Results:**

We discovered diverse expression pattern among IAPs, varied spectrum of apoptosis regulations through IAPs and extensive regulations beyond apoptosis involving immune response, cell cycle, gene expression and DNA damage repair. Importantly, IAPs were strong prognostic factors for patient survival and tumor stage in several tumor types including brain, liver, kidney, breast and lung cancer. Further, several IAPs were found to be predictive of sensitivity to BCL-2 inhibitors (BIRC3, BIRC5, BIRC6, and BIRC7) as well as RIPK1 inhibitors (BIRC3 and BIRC6).

**Conclusion:**

Together, our work revealed the landscape of regulations, prognostic utilities and therapeutic relevance of IAPs across multiple tumor types.

## Background

Inhibitors of apoptosis proteins (IAPs) are a family of regulators controlling multiple biological pathways involving various physiologic and pathologic conditions. To date, eight mammalian IAPs have been identified: BIRC1 (NAIP/NLRB), BIRC2 (cellular IAP1/cIAP1/human IAP2), BIRC3 (cellular IAP2/cIAP2/human IAP1), BIRC4 (X-linked IAP/XIAP), BIRC5 (survivin), BIRC6 (apollon/BRUCE), BIRC7 (livin/melanoma-IAP, also called ML-IAP/KIAP), and BIRC8 (testis-specific IAP/Ts-IAP/hILP-2) [1]. The defining characteristic of IAPs is the existence of the BIR (baculovirus IAP repeat) domain. In addition to the BIR domain, IAPs also contain other important domains including RING (C-terminal Ring zinc-finger domain), CARD (caspase recruitment domain) and UBC (C-terminal ubiquitin-conjugating domain) as depicted in Additional file 1: Figure S1.

IAPs impose apoptosis regulation through three well-characterized apoptosis pathways: (1) the extrinsic pathway, also known as the death receptor pathway, which activates caspase-8 and further propagates the apoptosis signal to effector caspases including caspase-3 upon the stimulation of death receptors; (2) the intrinsic pathway of apoptosis, also called the mitochondrial pathway, which starts with the release of apoptogenic factors such as cytochrome c into the cytosol triggering activation of caspase-3; and (3) the execution phase of apoptosis, which controls the breakdown of cells leading to cell death. The functions of IAPs in apoptosis have been wildly investigated in human cancers as therapeutic targets for anti-cancer drug development. Besides the roles in apoptosis regulation, growing evidence has demonstrated that IAPs are more than just inhibitors of apoptosis proteins and they can regulate various biological processes beyond apoptosis including immune response, cellular stress, translation, transcription, cell proliferation, differentiation, motility and signal transduction [2]. Futher, recent studies also found that IAPs have opposing roles in cancer serving as both oncogenes and tumor suppressors [3]. However, these studies were sporadic and sometimes contradictory. A systematic investigation of the complexity of apoptosis regulations by IAPs and their therapeutic relevance across various human cancers would provide a consolidated view and thus be invaluable for deepening our understandings.

The Cancer Genome Atlas (TCGA) is a large collection of human tumor data containing nearly 10,000 tumor patients from 32 different tumor types [4]. TCGA serves as an open repository of large-scale genome sequencing and multi-modal molecular profilings of human cancers. In addition to the TCGA patient data, the Genomics of Drug Sensitivity in Cancer (GDSC) database further provides large scale molecular characterization and drug profiling data for more than a thousand cell lines [5]. The emergence of such large scale genomic data for both patient and cancer cell lines greatly facilites multiple integrated genomic investigations across multiple cancers [6].

In this work, we leveraged the TCGA data to comprehensively characterize the global expression pattern of 7 IAPs across 32 types of human cancers. We investigated the co-expression pattern among IAPs demonstrating both similarity and differences in terms of expression dynamics, which served as the foundation of differential IAPs regulation in various pathways. We further identified several candidate miRNAs regulating IAPs, some of which had been experimentally validated in previous studies. For clinical translations, we found IAPs were strong prognostic markers for patient survival and tumor stage, with BIRC5 being the most potent biomarker for patient survival in multiple tumor types. Additionally, we found that IAPs predicted chemosensitivity of several anti-cancer drugs, further demonstrating the great potential of IAPs for therapeutics development.

## Results

### Global expression patterns among IAPs

**Fig. 1:**
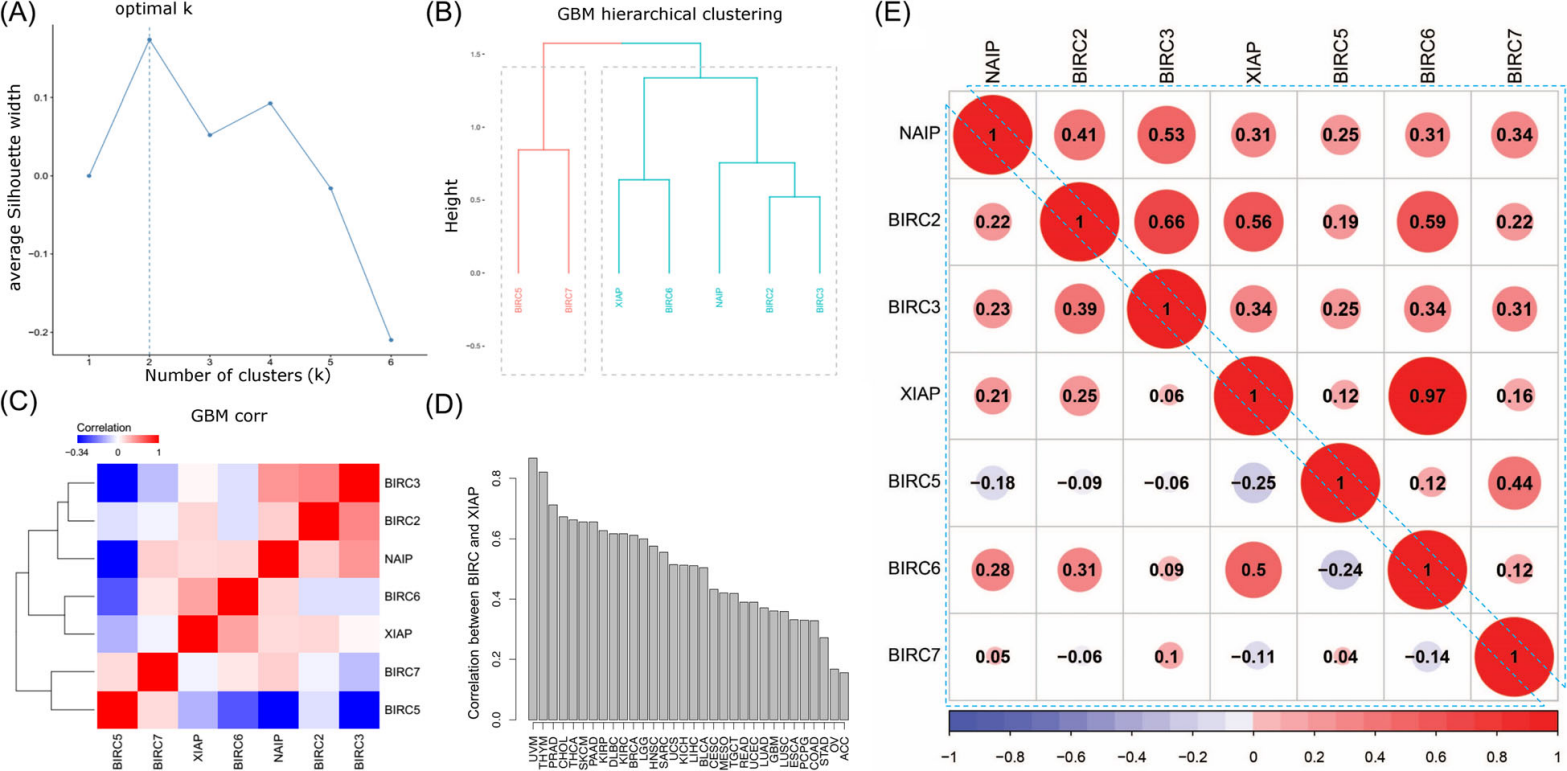
Global Expression Pattern of IAPs. The 7 IAPs have distinct expression pattern across 32 cancer types. Using GBM as an example, hierarchical clustering (**b**) using Ward’s linkage and correlation-based distance identified 2 clusters based on the criteria of maximum average Silhouette width (**a**). **c** Divergent correlation among the 7 IAPs in GBM. **d** Example association between XIAP and BIRC6 among 32 cancer types. All these correlations are statistically significant with *p* < 0.001. **e** A global view of the similarity of IAP expression patterns. The upper right triangle shows the frequency of any pair of IAPs being clustered together among 32 cancers. The lower left triangle shows the average correlation between any 2 pair of IAPs. Circle size indicate magnitude (frequency or correlation). Color indicates correlation direction, with red for positive correlation, blue for negative correlation and values indicated in the color legend. All correlations are computed using Spearman rank correlation

As a gene family, members of IAPs are characterized by the presence of one to three BIR domains and optionally a RING and CARD domain [7]. Despite of the similarity of gene structure, the gene expression pattern of IAPs as a whole is rarely studied. To systematically investigate the co-expression pattern of IAPs which forms the basis of orchestrated apoptosis regulation, we employed hierarchical clustering of these genes across 32 human cancers consisting of 9714 tumor samples (data summary provided in Additional file 6: Table S1) and catalogued the frequency of any gene pair being clustered together. We observed that except BIRC5 and BIRC7, there were broad expression similarity among IAPs (Fig. 1). For example, BIRC2 was clustered with BIRC3, XIAP and BIRC6 in more than 50% of the cancers. The strongest coherence was observed between XIAP and BIRC6 which were clustered together in 97% of the cancers. This was further confirmed by Spearman’s rank correlation analysis which quantified the actual strength of co-expression. XIAP and BIRC6 was strongly co-expressed with an average correlation over 0.5 (Fig. 1d and e). There was moderate co-expression among the NAIP-BIRC2-BIRC3-XIAP tetrad.

Unlike other IAPs, BIRC5 and BIRC7 had weak or even negative correlation with others (Fig. 1e). For example, BIRC5 showed moderate anti-correlation with XIAP, NAIP, and BIRC6 while BIRC7 showed weak anti-correlation with BIRC6 (Additional file 2: Figure S2A, Fig. 1e). Between BIRC5 and BIRC7, both positive and negative correlations were observed across the 32 cancers, demonstrating a histology dependent co-expression pattern (Additional file 2: Figure S2B).

Our global co-expression analysis of IAPs revealed distinct expression pattern of BIRC5 and BIRC7 as well as the synchronized expression for NAIP, BIRC2, BIRC3, XIAP and BIRC6. The demonstrated complexities of IAPs at the expression level provided immense possibilities for apoptosis regulation and potential mechanisms of oncogenesis from aberrant expression of IAPs.

### Spectrum of apoptosis regulations by IAPs

**Fig. 2:**
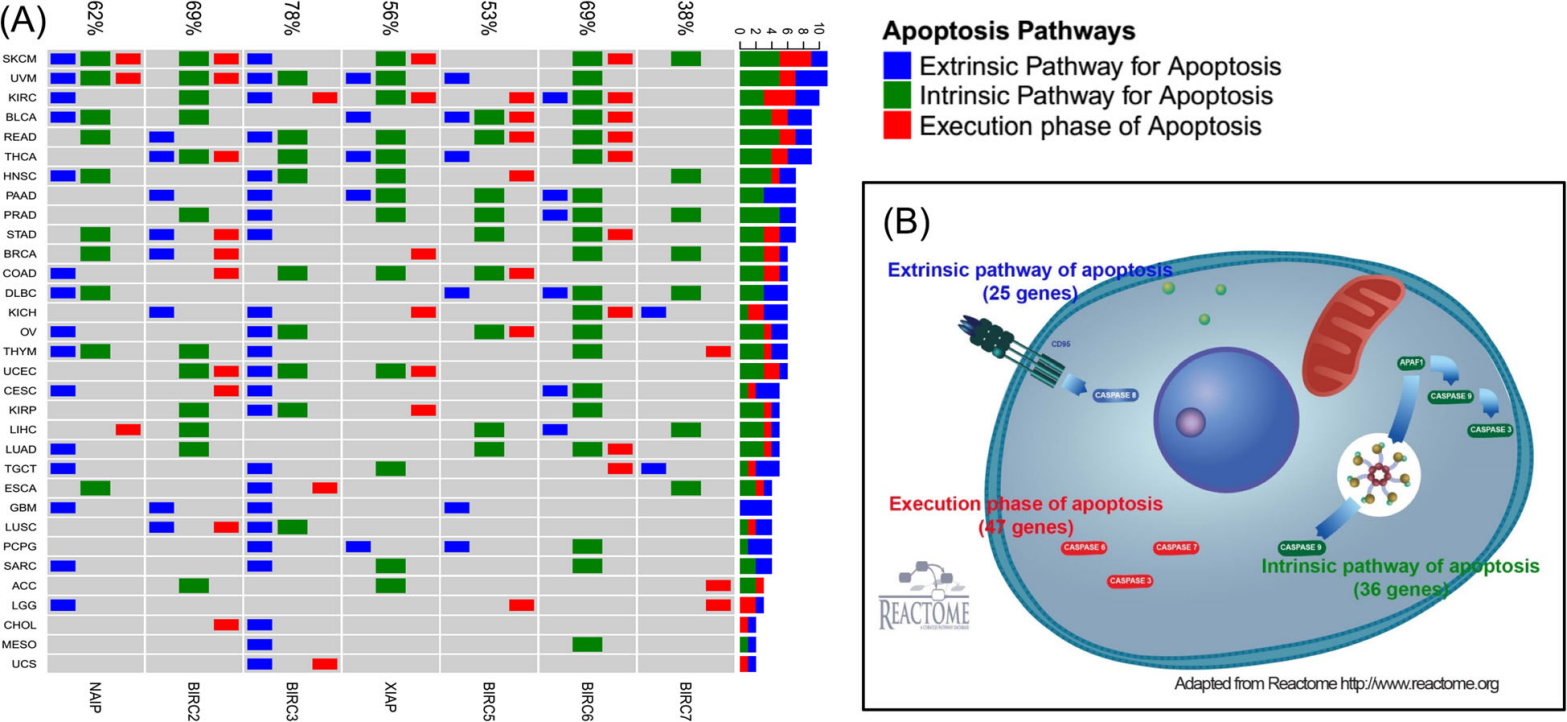
Spectrum of Apoptosis Regulations By IAPs. Gene set enrichment analysis identified different mechanisms for apoptosis regulations by different IAPs in different cancers (**a**) through three major apoptosis pathways including the extrinsic pathway of apoptosis (25 genes), the intrinsic pathway of apoptosis (36 genes) and the execution phase of apoptosis (47 genes) as depicted in (**b**). Percentage value on top of (**a**) summarized the proportion of cancers with at least one apoptosis pathways being regulated by a given IAP. The right hand bars on (**a**) summarized number of apoptosis pathways being regulated in each cancer. The intrinsic pathway of apoptosis (35.7%) as well as the extrinsic pathway of apoptosis (29.0%) were more frequently regulated by IAPs than the execution phase of apoptosis (18.3%) across all cancers. Here percentages were computed across all cancer-IAP regulation pairs. The frequency of apoptosis pathway regulations varied among different IAPs, with BIRC3 and BIRC6 both regulating 36.4% of cancers while BIRC7 only regulating 12.5% of cancers through at least one of the apoptosis pathways. Enriched pathways were identified with adjusted *p* value less than 0.05

IAPs are best known for their ability to regulate apoptosis. In an effort to systematically investigate the spectrum of apoptosis regulations by IAPs across various human cancers, we performed gene set enrichment analysis [8] using high quality pathways manually curated by experts provided by the Reactome database [9]. A total of 25 genes in the extrinsic pathway of apoptosis, 36 genes in the intrinsic pathway of apoptosis, and 47 genes in the execution phase of apoptosis were extracted (Fig. 2). Genes in these apopotosis pathways are provided in Additional file 8: Table S3.

Overall, the intrinsic pathway of apoptosis (35.7%) as well as the extrinsic pathway of apoptosis (29.0%) were more frequently regulated by IAPs than the execution phase of apoptosis (18.3%) across all cancers (proportion test *P* = 0.00018, Fig. 2a). The frequency of apoptosis pathway regulations varied among different IAPs, with BIRC3 and BIRC6 both regulating 36.4% of cancers while BIRC7 only regulating 12.5% of cancers in at least one of the apoptosis pathways (overall proportion test *P* = 0.0022). Mechanisms of apoptosis regulations through IAPs also differed. In particular, BIRC3 and NAIP mostly focused on the extrinsic pathway of apoptosis (average frequency of 59.4%) while BIRC6 and XIAP were heavily involved in the intrinsic pathway of apoptosis (average frequency of 48.4%). In contrast, there seemed to be a balanced distribution of the three different pathways for BIRC2 and BIRC5. The varied preferences of IAPs on regulating apoptosis pathways might due to the differential binding specificity to different caspases [10].

Importantly, regulated apoptosis pathways through IAPs also varied among different cancers, representing the histology-dependent functionalities of IAPs (Fig. 2a). For example, melanomas including skin cutaneous melanoma (SKCM) and uveal melanoma (UVM) had the most apoptosis regulations by IAPs followed by kidney renal clear cell carcinoma (KIRC). Adrenocortical carcinoma (ACC) and brain lower grade glioma (LGG) had the least apoptosis regulations through IAPs. Interestingly, cancers of similar tissue of origin had closer similarity in the pattern of apoptosis regulations such as skin cancers (SKCM, UVM), kidney cancers (KIRCH, KIRP) and brain cancers (LGG, GBM). The dissimilarity as well as commonality of apoptosis regulations revealed by our analysis highlighted the complexity of IAPs in regulating apoptosis and their importance in tumorigenesis.

### IAPs preserved extensive regulations beyond apoptosis

**Fig. 3:**
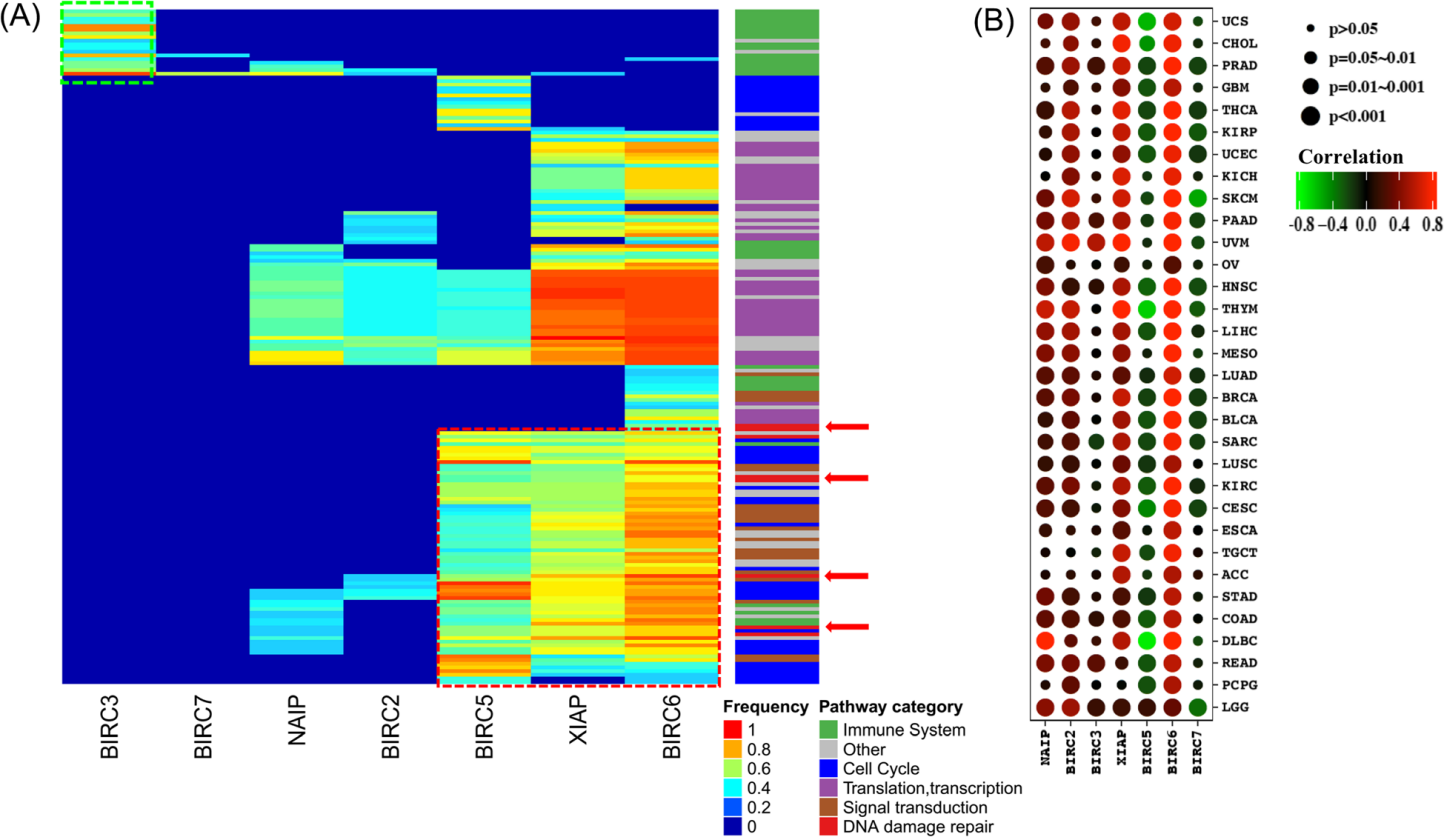
Global Pathways Regulated By IAPs. **a** Our analysis revealed extensive enriched pathways by IAPs beyond apoptosis including immune system, cell cycle, transcription and translation of genes, signal transduction, and DNA damage repair. Green dotted box highlighted the fact that BIRC3 mostly exerted regulations through the immune system containing interferon signaling, TCR signaling, PD-1 signaling and cytokine signaling pathways. The red dotted box highlighted that BIRC5, BIRC6 and XIAP were heavily involved in cell cycle pathways, signal transduction and DNA damage repair (red arrows). Additionally, NAIP, BIRC2, BIRC5, XIAP and BIRC6 were heavily involved in transcription and translation of genes. **b** Orchestrated REST and IAPs expression. BIRC5 and BIRC7 were negatively correlated with REST expression while other IAPs were positively correlated with REST expression in multiple cancers. For example, BIRC6 and XIAP were significantly positively correlated with REST expression across all cancer types except XIAP in PCPG. All of the 7 IAPs were significantly associated with REST in 5 cancer types, including PRAD, PAAD, HNSC, SARC, LGG. Each of the cancer types had at least 2 IAPs that correlated with REST expression

In addition to apoptosis regulations, our analysis unraveled extensive enriched pathways associated with the expression of IAPs involving immune system, cell cycle, transcription and translation of genes, signal transduction, and DNA damage repair (Fig. 3a). In particular, BIRC3 mostly exerted regulations through the immune system related pathways such as interferon signaling, TCR signaling, PD-1 signaling and cytokine signaling. Remarkably, cytokine signaling and interferon signaling regulated by BIRC3 were enriched in more than 80% of the cancers. Except BIRC3 and BIRC7, the other five IAPs were involved in transcription and translation of genes. Our result was in line with previous findings that IAPs exerted regulation of gene expression as well as immune system indirectly through the regulation of NF-κB signaling which was an important regulator of gene transcription involved in cell survival, differentiation and proliferation [10].

Further, IAPs were also highly associated with RE1-silencing transcription factor (REST) expression, an important factor for cancer progression and metastasis [11, 12] (Fig. 3b). REST had been reported as a master regulator in cancer development. However, few studies investigated the relationship between REST and IAPs. Our results revealed that REST and IAPs expression had been tightly correlated. BIRC5 and BIRC7 were significantly negatively correlated with REST expression, whereas other IAPs were positively correlated with REST expression in multiple cancers. In particular, BIRC6 and XIAP were positively correlated with REST expression across all cancer types except XIAP in PCPG. Since REST was a master negative regulator, we speculate BIRC5 and BIRC7 were downstream of REST, whereas NAIP, BIRC2, BIRC3, XIAP and BIRC6 were located upstream of REST. These data suggest IAPs had strong relationship with REST expression, both of which played important roles in tumorigenesis.

We also found that BIRC5, BIRC6 and XIAP were heavily involved in cell cycle related pathways such as M/G1 transition, G1/S transition, G2/M checkpoints, and DNA replication. Additionally, BIRC5, BIRC6, XIAP and BIRC2 were found to be involved in DNA damage repair. This was in line with previous findings that BIRC2 and XIAP were intermediates of DNA damage response and NF-κB signaling [13]. There were sporadic regulations exerted by BIRC7, with the majority of enriched pathways shared by less than one third of the cancers. Our discovery confirmed previous findings that IAPs were more than just inhibitors of apoptosis and their functions extended to a wide variety of cellular functions including cell cycle, cell division and signal transduction [14].

### Oncogenic and anti-oncogenic roles of IAPs

**Fig. 4:**
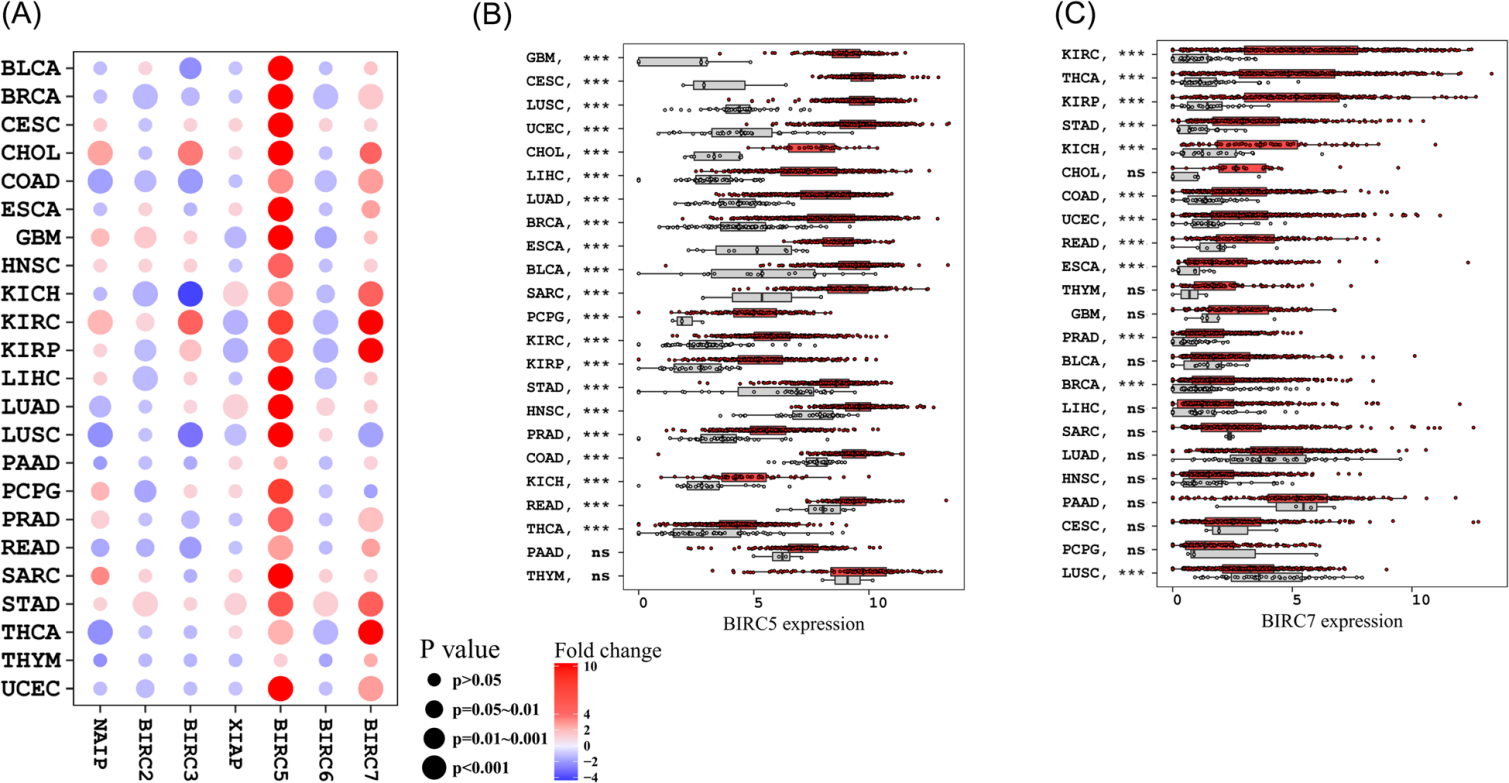
Oncogenic and Anti-oncogenic Roles of IAPs. Global comparison of IAPs expression between tumor and adjacent normal tissues across different cancers demonstrated that IAPs had extensive oncogenic or anti-oncogenic roles (**a**). A detailed visualization of BIRC5 and BIRC7 was provided in (**b**) and (**c**). In particular, BIRC5 was significantly overexpressed in 94% of the cancers followed by BIRC7 which was overexpressed in 66% of the cancers. Unlike BIRC5 and BIRC7 which demonstrated a strong oncogenic role, BIRC6 was down-regulated in eight human cancers compared to adjacent normal tissue. The differential expression of NAIP, BIRC2, BIRC3 and XIAP were cancer-type specific

To systematically evaluate how IAPs might contribute to tumorigenesis, we performed a global comparison of IAPs expression between tumor and adjacent normal tissues across 32 cancers. Remarkably, BIRC5 (survivin) was significantly overexpressed in 94% of the cancers followed by BIRC7 which was overexpressed in 66% of the cancers (Fig. 4). Interestingly, BIRC5 was significantly over-expressed in 21 human cancers. Unlike BIRC5 and BIRC7 which demonstrated a strong oncogenic role, BIRC6 tended to function as a tumor suppressor since it was down-regulated in eight human cancers. This was in line with previous findings where BIRC6 expression in acute myeloid leukemia patients was significantly lower than normal tissue (https://www.ncbi.nlm.nih.gov/pmc/articles/PMC3514096/). Other IAPs were also dysregulated and when dysregulated, both up-regulation and down-regulation were observed depending on cancer types (Fig. 4). For example, BIRC3 was up-regulated in CHOL, KIRC, KIRP while down-regulated in COAD, KICH, LUSC, and READ. Sample size as well as detailed analysis results were provided in Additional files 9 and 10: Table S4 and Table S5. Our analysis reinforced previous findings that many IAPs functioned as oncogenes [15].

### Regulations of IAPs by miRNAs

Since miRNA had been identified as important regulators of gene expression, we sought to evaluate how miRNA might affect the expression of IAPs. Through our comprehensive cross-cancer bioinformatic analysis, we identified a large number of miRNAs regulating IAPs across the cancers (Additional file 3: Figure S3). For example, BIRC5 was significantly anti-correlated with miR-101, miR-664a, miR-29c, miR-30a, miR-125 and miR-139 expression in over 40% of the cancers. BIRC3 was significantly anti-correlated with miR-96, miR-130, miR-149, miR-183, miR-1296, and miR-3200 in over 35% of the cancers; BIRC6 was significantly anti-correlated with miR-22, miR-26, miR-339, miR-362, and miR-3613 in over 30% of the cancers; and XIAP was significantly anti-correlated with miR-17, miR-18, and miR-24 in more than 30% of the cancers. Importantly, the interactions between the top anti-correlated miRNAs (see miR-101, miR-664a and miR-22 in Additional file 3: Figure S3) and IAPs were experimentally verified in previous studies, which further supported the regulatory roles of these miRNAs [16–18]. For the other three genes – BIRC7, NAIP and BIRC2, there were much less negatively correlated miRNAs. Considering that the three genes were not prone to interact with miRNAs [19], we speculated that their dysregulation was more likely induced by other factors.

### IAPs as prognostic factors for patient survival

**Fig. 5:**
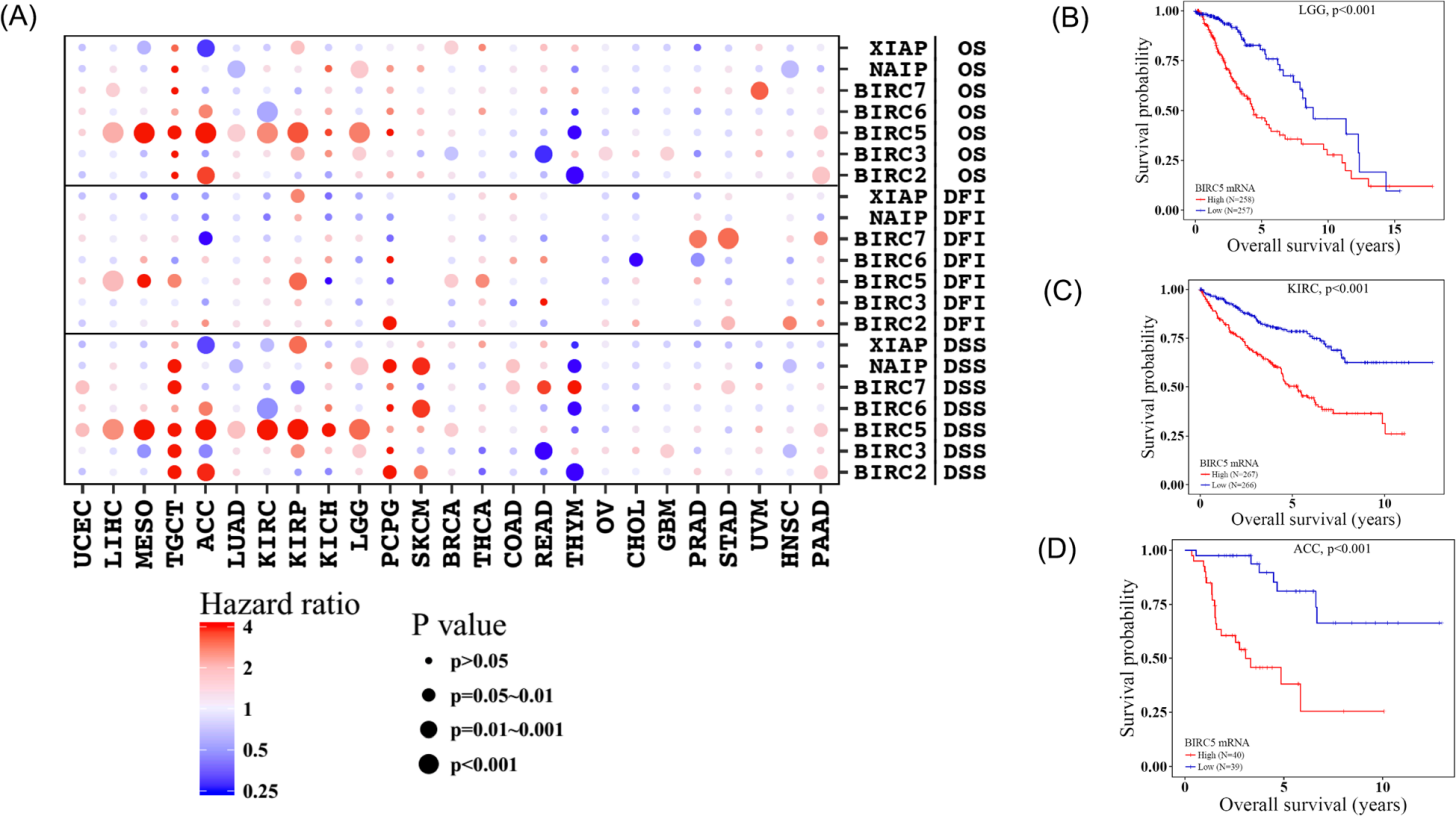
Global Prognostic Values of IAPs for patient survival. IAPs differed in the prognostic values among different cancers. Higher IAPs expression were associated with shorter survival most of the time, but there were also cases where higher expression was significantly associated with longer survival. Among all the IAPs, BIRC5 emerged as the most potent marker for survival with broad spectrum of prognostic utility across multiple cancers. (**a**). Kaplan-Meier curves showing high BIRC5 expression was associated with poor overall survival in LGG (**b**), KIRC (**c**) and ACC (**d**)

Evasion of apoptosis through dysregulated IAPs might led to uncontrolled cell growth and poor prognosis. To comprehensively evaluate the prognostic relevance of IAPs for patient survival across diverse cancers, we applied log-rank test to associate expression levels of different IAPs to three types of survival time including overall survival (OS), disease specific survival (DSS), and disease free interval (DFI). BIRC5 emerged as the most powerful prognostic biomarker for patient survival. In particular, higher BIRC5 expression was significantly associated with worse OS and DSS in LIHC, MESO, TGCT, ACC, LUAD, KIRC, KIRP, LGG and PAAD as well as worse DFI in LIHC, MESO, TGCT, KIRP, THCA and BRCA (Fig. 5). Higher BIRC5 expression was predominantly associated with worse survival across different cancers with one exception in THYM where higher BIRC5 was associated with longer overall survival.

In addition to BIRC5, other members of IAPs also had significant associations with survival and presented both positive and negative associations. For example, higher XIAP expression was associated with worse OS in BRCA, worse OS/DSS/DFI in KIRP but better OS/DSS in ACC and better OS in MESO (Fig. 5). This was consistent with previous studies which demonstrated controversial prognostic role of XIAP for cancer patients with increased XIAP expression predicting shorter overall survival in acute myeloid leukemia (AML) but longer overall survival in cervical carcinoma [20–22], prostate cancer [23] and lung cancer [24]. Similarly, higher expression of BIRC2 was prognostic for worse survival in SKCM, PCPG, PAAD, HNSC, STAD, ACC, TGCT but better survival in THYM. Higher expression of NAIP was associated with worse survival in COAD, SKCM, PCPG, LGG, TGCT but better survival in HNSC, THYM, LUAD. In terms of cancers, UCS, DLBC, and ESCA survival was not associated with any of the IAPs. THYM survival was mostly negatively associated with IAPs expression while TGCT, PCPG and SKCM survival was mostly positively associated with IAPs expression (Fig. 5). The wide spectrum of associations between IAPs expression and patient survival implied a more complicated role of IAPs in tumor biology than previously reported.

### IAPs as prognostic factors for tumor stage

**Fig. 6:**
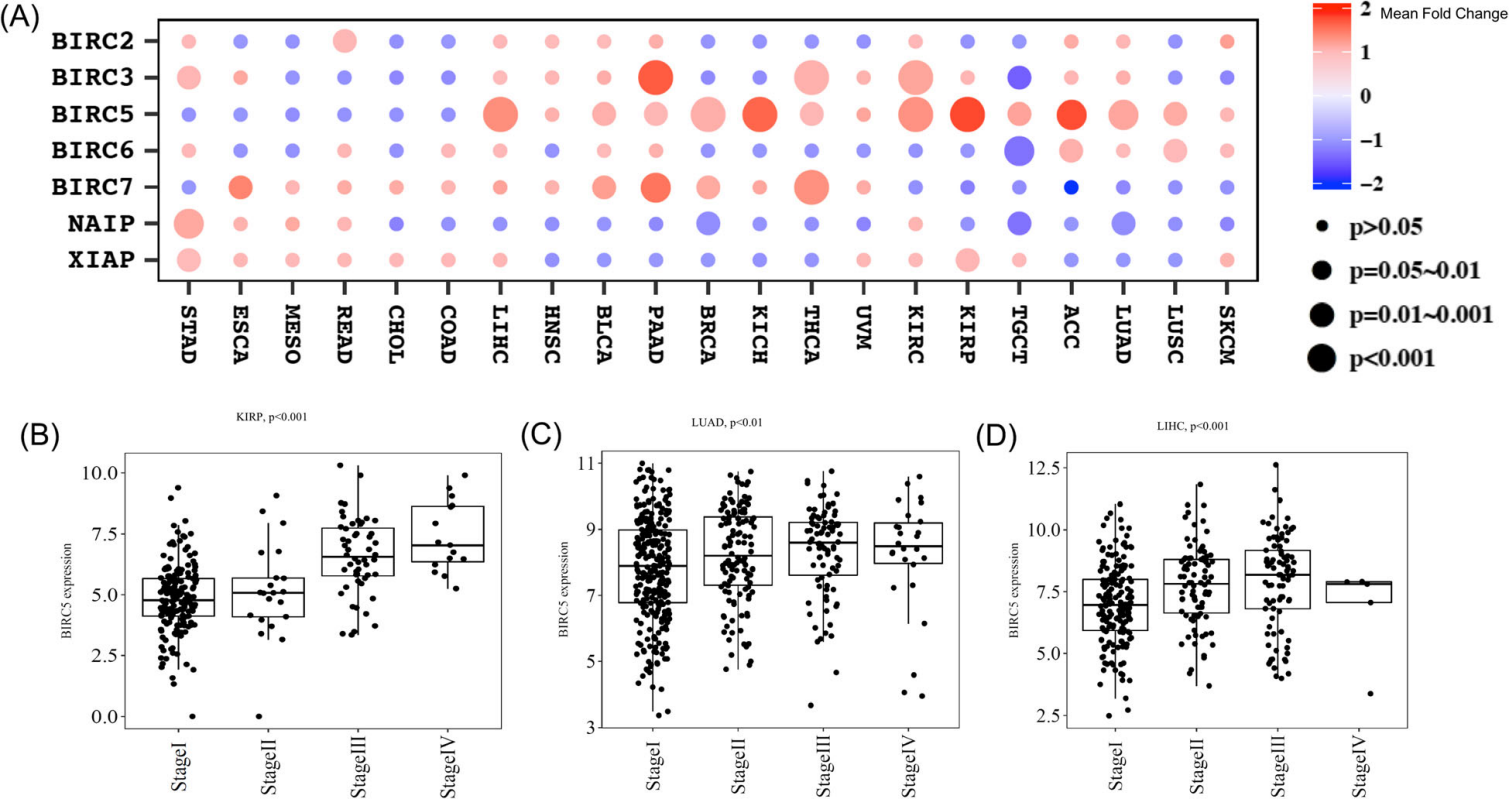
Prognostic Value of IAPs for tumor stage. 32 significant associations (*p* < 0.05) were identified between the 7 IAPs expression and different cancer types using ANOVA test (**a**). BIRC5 accounted for 12 of the significant associations (38%), all of which were positive associations where increased expression was associated with more advanced tumor stage. Examples associations between BIRC5 expression and tumor stage in KIRP (**b**), LUAD (**c**) and LIHC(**d**)

Tumor staging determines the severity of cancer and the extent cancer has spread in the body and thus provides critical information for treatment plan. In order to evaluate the prognostic value of IAPs for tumor stage, we performed comprehensive hypothesis testing between IAPs expression and tumor stage across all cancers. A total of 32 significant associations (defined as ANOVA test *p* < 0.05, corresponding false discovery rate = 0.09 after adjusting for multiple hypothesis testing) were identified between the 7 IAPs expression and 32 cancer types (Fig. 6). 27 (84%) of these were positive associations where higher IAPs expression was associated with higher tumor stage. Among all the IAPs, BIRC5 was the most predictive gene of tumor stage accounting for 12 of the significant associations (38%), all of which were positive associations which agreed with previous publications [25–27]. BIRC3 and BIRC7 were the second most predictive genes each associated with tumor stage in five cancer types. Surprisingly, there were five negative associations between IAPs expression and tumor stage, with three in TGCT, one in BRCA and one in LUAD (Additional file 4: Figure S4). To our best knowledge, there were no publications reporting similar negative associations and thus these findings from our comprehensive analysis warranted further investigations.

### IAPs and chemosensitivity

**Fig. 7:**
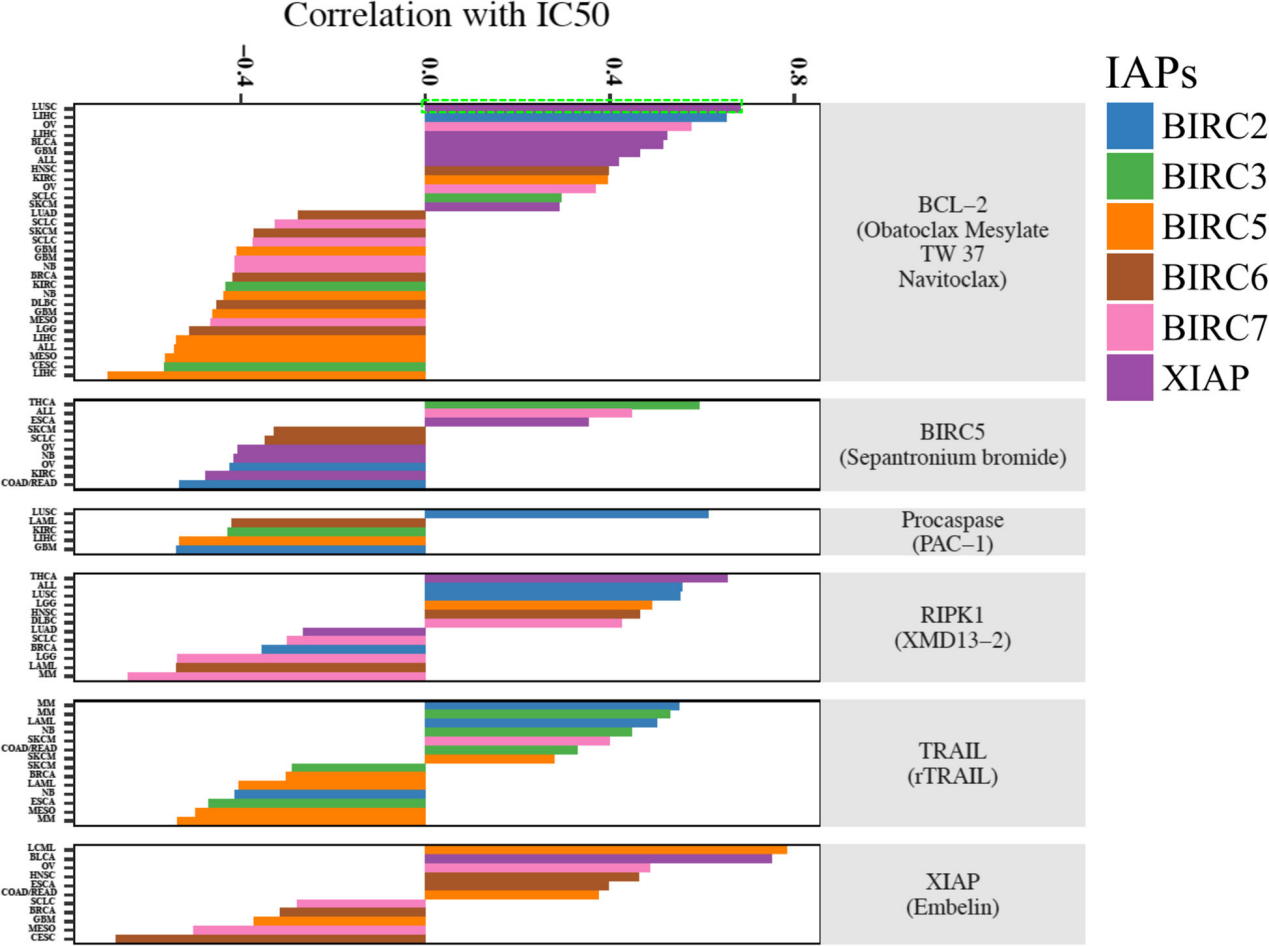
IAPs Determine Sensitivity to Apoptosis Inhibitors from preclinical cell line data. From the Genomics of Drug Sensitivity in Cancer (GDSC) database, we identified 8 drugs targeting the apoptosis pathway involving targets of BCL-2, BIRC5, Procaspases, RIPK1, TRAIL, and XIAP. The spectrum of association between IAPs expression and apoptosis targets was quite diverse, with the expression of BIRC3, BIRC5, BIRC6, BIRC7 mostly associated with sensitivity to BCL-2 inhibitors while the expression of XIAP and BIRC2 mostly associated with resistance. As an example, green dotted box represented LUSC cell lines with high XIAP expression being resistant to obatoclax mesylate, a BCL-2 inhibitor. Red dotted box represented obatoclax mesylate was sensitive to LIHC cell line with high BIRC5 expression

After observing the strong oncogenic role of IAPs and their association with poor survival, we further evaluated if IAPs were able to predict chemosensitivity of drugs targeting apoptosis. Using the Genomics of Drug Sensitivity in Cancer (GDSC) database for drug sensitivity and gene expression, we identified 8 drugs targeting the apoptosis pathway involving targets of BCL-2, BIRC5, Procaspases, RIPK1, TRAIL, and XIAP (Fig. 7). The detailed analysis result can be found in Additional file 11: Table S6. A wide spectrum of association pattern was observed between IAPs expression and different apoptosis targets. For example, the expression of BIRC3, BIRC5, BIRC6, and BIRC7 was mostly associated with sensitivity to BCL-2 inhibitors while the expression of XIAP and BIRC2 was associated with resistance. Interestingly, BIRC5 expression was not associated with sensitivity to the BIRC5 inhibitor Sepantronium bromide (also called YM155). Instead, BIRC5 expression was mostly associated with sensitivity to BCL-2/TRAIL inhibitors and resistance to XIAP/RIPK1 inhibitors (Additional file 7: Table S2). In contrast, the expression of BIRC2 and BIRC6 was associated with sensitivity to BIRC5 inhibition. Previous studies found that BIRC3 facilitated cell survival by promoting RIP1 degradation [28]. Here we found that the expression of BIRC3 and BIRC6 was also associated with sensitivity to RIPK1 inhibition. There was also a cancer-specific effect on association between IAPs expression and drug sensitivity. In particular, drugs targeting RIPK1, TRAIL and XIAP had opposite correlations in different cancer types with the same IAP.

**Fig. 8:**
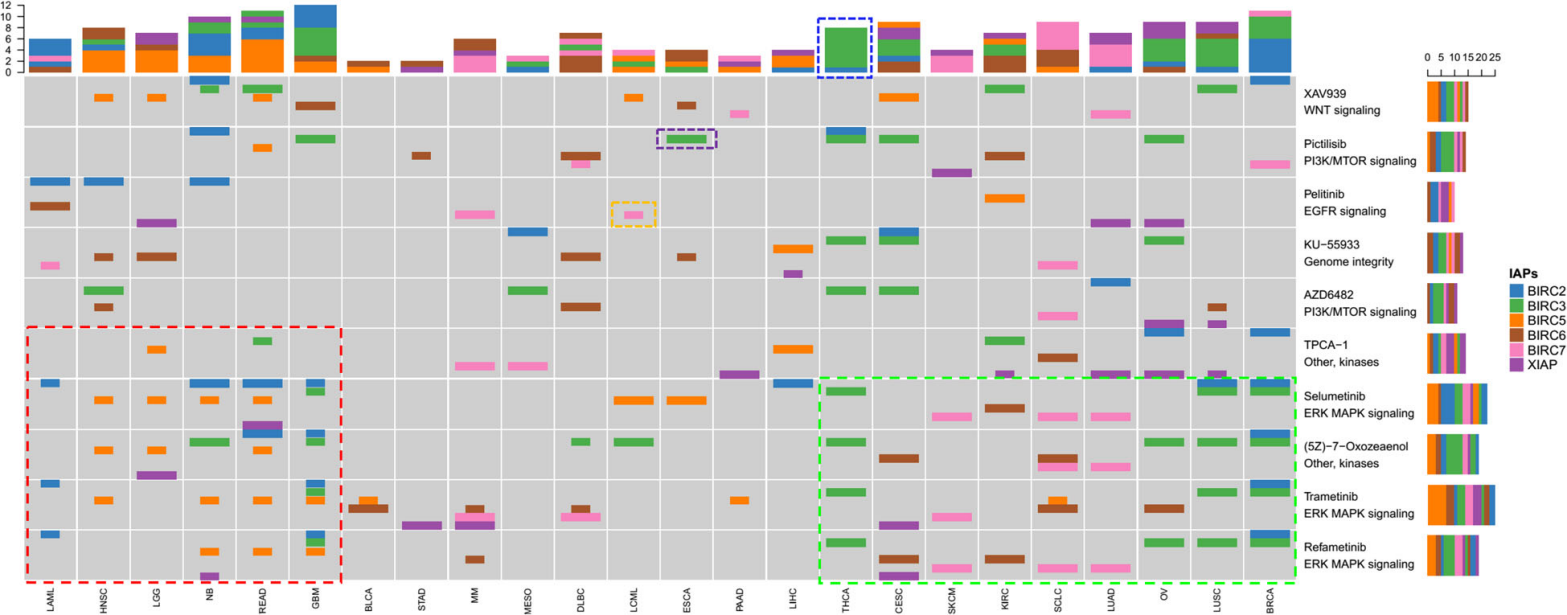
Global Landscape of Chemosensitivity Associated with IAPs using preclinical cell line data. Expanding our analysis to all the 250 drugs profiled by GDSC, we obtained a global landscape of chemosensitivity associated with IAPs expression. Focusing on the drugs that were most frequently associated with drug sensitivity, we identified 10 drugs targeting 5 major pathways including EGFR signaling, ERK MAPK signaling, genome integrity, PI3K/MTOR signaling, and WNT signaling. Narrower bars indicate positive correlation (higher expression predicting resistance). For example, yellow dotted bar represented LCML cell lines with high BIRC7 expression being resistant to Pelitinib, an inhibitor to EGFR signaling. Wider bars indicate negative correlation (higher expression predicting sensitivity). For example, purple dotted bar represented ESCA cell lines with high BIRC3 expression was sensitive to Pictilisib, an inhibitor to the PI3K/MTOR signaling pathway. There were six cancers (LGG, GBM, NB, LAML, HNSC, READ) on the left of the figure (red dotted box) where IAPs expression was mostly associated with drug resistance and nine cancers (SCLC, LUAD, LUSC, THCA, CESC, SKCM, KIRC, BRCA, OV) on the right (green dotted box) where IAPs expression was associated with drug sensitivity. Blue dotted box circling the top column bar summarized sensitivity to seven drugs when BIRC3 expression was high, and resistance to one drug when BIRC2 expression was high in THCA

We further expanded our analysis to all the drugs profiled by GDSC and obtained a global landscape of chemosensitivity associated with IAPs expression. We observed a wide spectrum of associations between IAPs expression and drug sensitivity. To have a focused view of the most significant associations, we examined drugs that were significantly associated with the expression of at least one IAP in at least 8 cancer types (32% of the cancers) which led to 10 drugs targeting 5 major pathways including EGFR signaling, ERK MAPK signaling, genome integrity, PI3K/MTOR signaling, and WNT signaling (Fig. 8). Additional associations with inhibitors of DNA replication, EGFR signaling, ERK MAPK signaling, Mitosis, PI3K/MTOR signaling, protein stability and degradation, and RTK signaling were provided in Additional file 5: Figure S5. The three drugs targeting the ERK MAPK signaling pathway immediately split the cancers into three groups: a group of 6 cancers (LGG, GBM, NB, LAML, HNSC, READ) where IAPs expression were associated with drug resistance (ERK-MAPKi resistance group), a group of 9 cancers (SCLC, LUAD, LUSC, THCA, CESC, SKCM, KIRC, BRCA, OV) whose IAPs expression were associated with drug sensitivity (ERK-MAPKi sensitive group), and a third group of 9 cancers which had presented weaker association between IAPs expression and drug sensitivity. Importantly, IAPs expression in the ERK-MAPKi resistance group including 3 types of brain cancers were associated with sensitivity to EGFR inhibitor, PI3K/MTOR inhibitor and WNT signaling inhibitors. Interestingly, BIRC5 previously found to be associated with sensitivity to apoptosis inhibitors was found to be mostly associated with drug resistance for drugs not targeting apoptosis. The expression of BIRC3 and BIRC7 demonstrated the most associations with drug sensitivity. Our global analysis of chemosensitivity suggest that IAPs may be good biomarkers for sensitivity of ERK-MAPK inhibitors in multiple cancers including 3 types of lung cancers and possible biomarkers of sensitivity to EGFR inhibitor, PI3K/MTOR inhibitor and WNT signaling inhibitor for cancers resistant to ERK-MAPK inhibition.

## Discussion

Chemotherapy resistance is a serious bottleneck of anticancer drug treatments. Fundamentally, drug resistance occurs when malignant cells fail to die in response to therapy, in part due to the failure of apoptosis and caspase activation [29]. IAPs as antiapoptotic proteins block cell death and promote uncontrolled cell division resulting in tumor malignancy as well as treatment resistance [30]. Molecularly, IAPs inhibit apoptosis through three major pathways: [1] the intrinsic pathway, also called the mitochondrial pathway starting from the release of cytochrome c into the cytosol, [2] the extrinsic pathway, also called the death receptor pathway which activates caspase-8 and effector caspases, and [3] the execution phase of apoptosis, also called the Cross-Talk step that destructs the cell organelles [14].. With the completion of large scale genomic characterization of patient tumors (the TCGA project) and molecular characterization of cell lines coupled with high-throughput drug screening (the GDSC database), we conducted for the first time a systematic investigation of the co-expression pattern of IAPs, the regulation spectrum on apoptosis and other pathways, prognostic values on patient survival, and predictive utility of IAPs on drug sensitivity among diverse tumor types.

Our co-expression analysis identified commonalities as well as complexities of IAPs expression profiles using patient tumors which was more clinically relevant than immortalized cell lines. The first attempt to investigate co-expression pattern of IAPs was conducted with the National Cancer Institute panel of 60 human tumor cell lines (NCI60) [31]. XIAP and BIRC2 were found to express highly among different cancers while NAIP and BIRC3 were only selectively expressed in a few cell lines. Despite their limited sample size on 60 cell lines, their results were in line with our findings where XIAP and BIRC2 were clustered together in 56% of the tumors (fourth highest among all the pairs). Compared to the NCI60 analysis, we further revealed distinct expression pattern of BIRC5 and BIRC7 and synchronized expression for NAIP, BIRC2, BIRC3, XIAP and BIRC6 in multiple tumor types. The demonstrated complexities of IAPs expression provided vast possibilities for apoptosis regulation and potential mechanisms of oncogenesis resulting from aberrant expression of IAPs.

Our study also provided quantitative insights on the mechanisms of apoptosis regulations through IAPs across diverse tumor types. IAPs were previously reported to be potent regulators of both intrinsic and extrinsic apoptotic pathways [32]. Expanding on this, we found the intrinsic and extrinsic pathways of apoptosis were more frequently regulated by IAPs than the execution phase of apoptosis across all cancers. We also observed a histology-dependent pattern on apoptosis regulations where skin cancers (SKCM, UVM) had the most apoptosis regulations through IAPs followed by kidney renal clear cell carcinoma (KIRC). Adrenocortical carcinoma (ACC) and lower grade glioma (LGG) had the least apoptosis regulations through IAPs. The capacity of apoptosis regulations among IAPs also differed with BIRC3 and BIRC6 both regulating 36.4% of cancers while BIRC7 only regulating 12.5% of cancers in at least one of the apoptosis pathways. In addition, we found IAPs were also involved in immune response, cell cycle, transcription and translation of genes, signal transduction, and DNA damage repair. These discoveries confirmed and expanded previous findings that IAPs were more than just apoptosis regulators [2].

We also demonstrated therapeutic importance of IAPs through differential expression analysis as well as association with clinicopathological parameters. BIRC5 was the most overexpressed IAP across multiple tumors, consistent with previous studies showing its strong oncogenic roles across multiple cancers [33–35]. Higher BIRC5 expression was also strongly associated with worse patient survival and advanced tumor stage. The other IAPs also presented varied therapeutic potential in a histology dependent manner. These results suggested the prognostic value of IAPs was contextual dependent on cancer types as well as clinical outcomes demonstrating the great potential of personalized cancer therapy. Compared to cytotoxic chemotherapy, IAPs inhibitors were shown to be less toxic to normal cells [22] and preserved favorable safety profiles through clinical studies [36]. These together make IAPs targeting a promising approach for improved cancer therapy.

## Conclusions

We discovered diverse expression patterns among IAPs, their wide spectrum of apoptosis regulations as well as regulations beyond apoptosis involving immune response, cell cycle, gene expression and DNA damage repair. We also demonstrated IAPs were prognostic factors for patient survival and tumor stage in several tumor types. Finally, several IAPs (BIRC3, BIRC5, BIRC6, and BIRC7) were found to be predictive of sensitivity to BCL-2 inhibitors as well as RIPK1 inhibitors (BIRC3 and BIRC6). Together, our work demonstrated the great potential of targeted therapy using IAPs.

## Materials and methods

### Data collection

We downloaded the TCGA gene expression data using the Firehose command line tool (https://confluence.broadinstitute.org/display/GDAC/Download). Normalized RNAseqV2 data with RSEM quantification [37] was used for all TCGA expression analysis. A total of 32 cancers with 9714 patients were downloaded. For copy number data, gene level results from GISTIC analysis [38] of the Affymetrix SNP 6.0 arrays were downloaded from Firehose. Similarly, normalized level 3 miRNA expression data was downloaded from Firehose. The clinical data including tumor stage and survival time was downloaded from Genomic Data Commons Data Portal (https://portal.gdc.cancer.gov/).

For GDSC database, cell line information including tissue type and cell line names was downloaded from the COSMIC database (https://cancer.sanger.ac.uk/cosmic). IC50 values as well as normalized gene expression data for the cell lines were downloaded from the GDSC official site (https://www.cancerrxgene.org/). Cell line information was matched with both IC50 values and gene expression data using GDSC internal ID.

### Co-expression analysis of IAPs

To systematically investigate the co-expression pattern of the IAPs among all the cancer types, hierarchical clustering analysis with automatic cluster size selection was used. In particular, correlation-based distance and Ward’s linkage was used to construct the hierarchical clustering and optimal cluster size was identified by maximizing the average Silhouette width among the clusters. The frequency of IAPs being clustered together was calculated as the ratio of the number of times of any two IAPs being clustered together based on the optimal cluster size divided by total number of cancers (32 in this case). We also quantified the actual strength of co-expression between any two IAPs using Spearman’s rank correlation.

### Pathway enrichment analysis

clusterProfiler and CAsubtype [39, 40] were used for gene set enrichment analysis. For each of the IAPs, we computed the spearman rank correlation with all other mRNAs resulting in a ranked list of genes. Based on this ranked gene list, clusterProfiler implemented a permutation test [8] to assess the significance of the enrichment for each of the manually curated pathways in the Reactome database. To account for multiple hypothesis testing, adjusted *p* values were calculated using the Benjamini-Hochberg procedure which controlled for the false discovery rate [41]. Enriched apoptosis pathways were identified with adjusted p value less than 0.05. Similarly, global pathways regulated by IAPs were identified with adjusted p value less than 0.01.

### Statistical analysis

To assess the association between miRNA and IAPs expression, we computed Spearman rank correlation between each IAP and miRNA within each cancer type. To search for miRNAs that might target IAPs, we identified significant anti-correlations with Spearman rank correlation less than − 0.2 and adjusted p value less than 0.05. This criterion not only ensured statistical significance, but also captured enough strength of the correlation.

Student’s t-test was used to identify differential expression between tumor and adjacent normal tissues. Logrank test was used to compare survival difference between low and high expression groups defined by median IAPs expression. ANOVA test was used to compare expression differences among different tumor stages. Spearman rank correlation was used to assess association between drug sensitivity and IAPs expression. All statistical analysis was performed using the R software [42].

## Supplementary information

**Additional file 1: Figure S1.** Domain structure of IAP protein family. Existence of at least one BIR domain is the defining characteristic of IAP family. Several IAPs also contain a RING-zinc finger domain (BIRC2, BIRC3, BIRC5, BIRC7 and BIRC8) at the carboxy terminus with autoubiquitination and degradation activity. BIRC2 and BIRC3 both have a CARD domain between the BIR domains and the RING domain. BIRC6 is unique containing an UBC domain. BIR: baculovirus IAP repeat; CARD: caspase recruitment domain; RING, C-terminal Ring zinc-finger domain; UBC, C-terminal ubiquitin-conjugating domain.

**Additional file 2: Figure S2.** Example Co-expression Between IAPs. BIRC5 mostly showed anti-correlation with NAIP expression across different cancers (A). Between BIRC5 and BIRC7, both positive and negative correlations were observed across the 32 cancers (B).

**Additional file 3: Figure S3.** Regulation of IAPs by miRNAs. For each miRNA (rows) in a given IAP gene (columns), we compute frequency among 32 cancers that have significant anti-correlation between miRNA and IAP gene expression (correlation < − 0.2; adjusted *p* value < 0.05).

**Additional file 4: Figure S4.** Example Negative Associations Between IAPs expression and Tumor Stage. (A), (B) and (C) were from TGCT, (D) was from BRCA and (E) was from LUAD.

**Additional file 5: Figure S5.** IAPs Determine Sensitivity to Other Inhibitors.

**Additional file 6: Table S1.** TCGA Data Summary and Cancer Acronyms.

**Additional file 7: Table S2.** BIRC5 Determines Sensitivity to IAP inhibition.

**Additional file 8: Table S3.** Apoptosis pathway genes.

**Additional file 9: Table S4.** Sample size for TCGA tumor vs adjacent normal comparisons.

**Additional file 10: Table S5.** Results of TCGA tumor vs adjacent normal comparisons.

**Additional file 11: Table S6.** Results of Sensitivity analysis for Apoptosis inhibitors.

## Data Availability

All data analyzed were publicly available (see Methods).

## Abbreviations

AML: Acute myeloid leukemia
DFI: Disease free interval
DSS: Disease specific survival
GDSC: Genomics of drug sensitivity in cancer
IAP: Inhibitors of apoptosis protein
OS: Overall survival
REST: RE1-silencing transcription factor
TCGA: The Cancer Genome Atlas
