# Supplementary figures and images for: Comprehensive molecular characterization of inhibitors of apoptosis proteins (IAPs) for therapeutic targeting in cancer
Source: BMC Med Genomics. 2020 Jan 21;13:7. doi: 10.1186/s12920-020-0661-x (PMC6975060; doi:10.1186/s12920-020-0661-x)

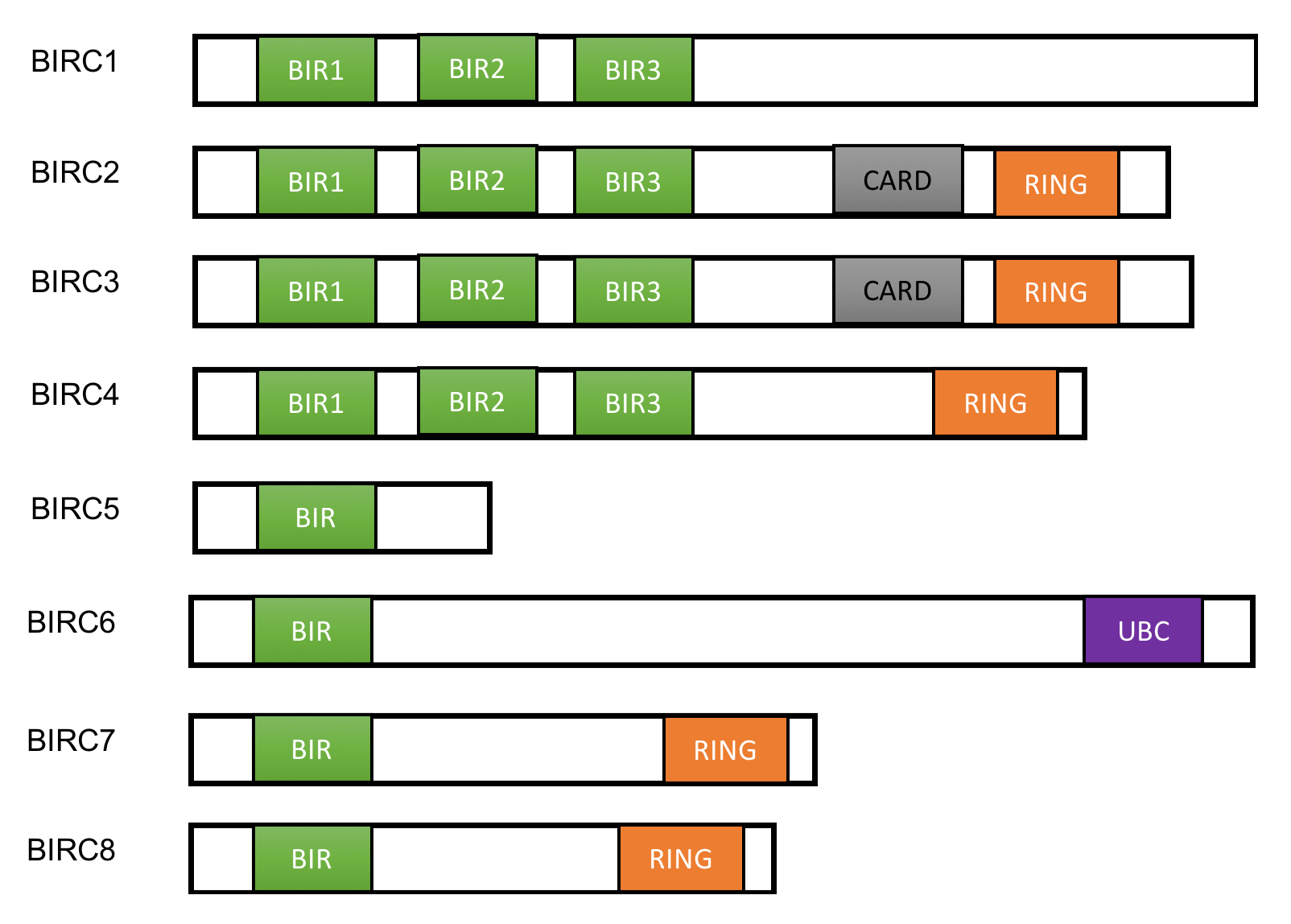

Supplement: Supplementary file 1 — Additional file 1: Figure S1. Domain structure of IAP protein family. Existence of at least one BIR domain is the defining characteristic of IAP family. Several IAPs also contain a RING-zinc finger domain (BIRC2, BIRC3, BIRC5, BIRC7 and BIRC8) at the carboxy terminus with autoubiquitination and degradation activity. BIRC2 and BIRC3 both have a CARD domain between the BIR domains and the RING domain. BIRC6 is unique containing an UBC domain. BIR: baculovirus IAP repeat; CARD: caspase recruitment domain; RING, C-terminal Ring zinc-finger domain; UBC, C-terminal ubiquitin-conjugating domain. [file 12920_2020_661_MOESM1_ESM.png]

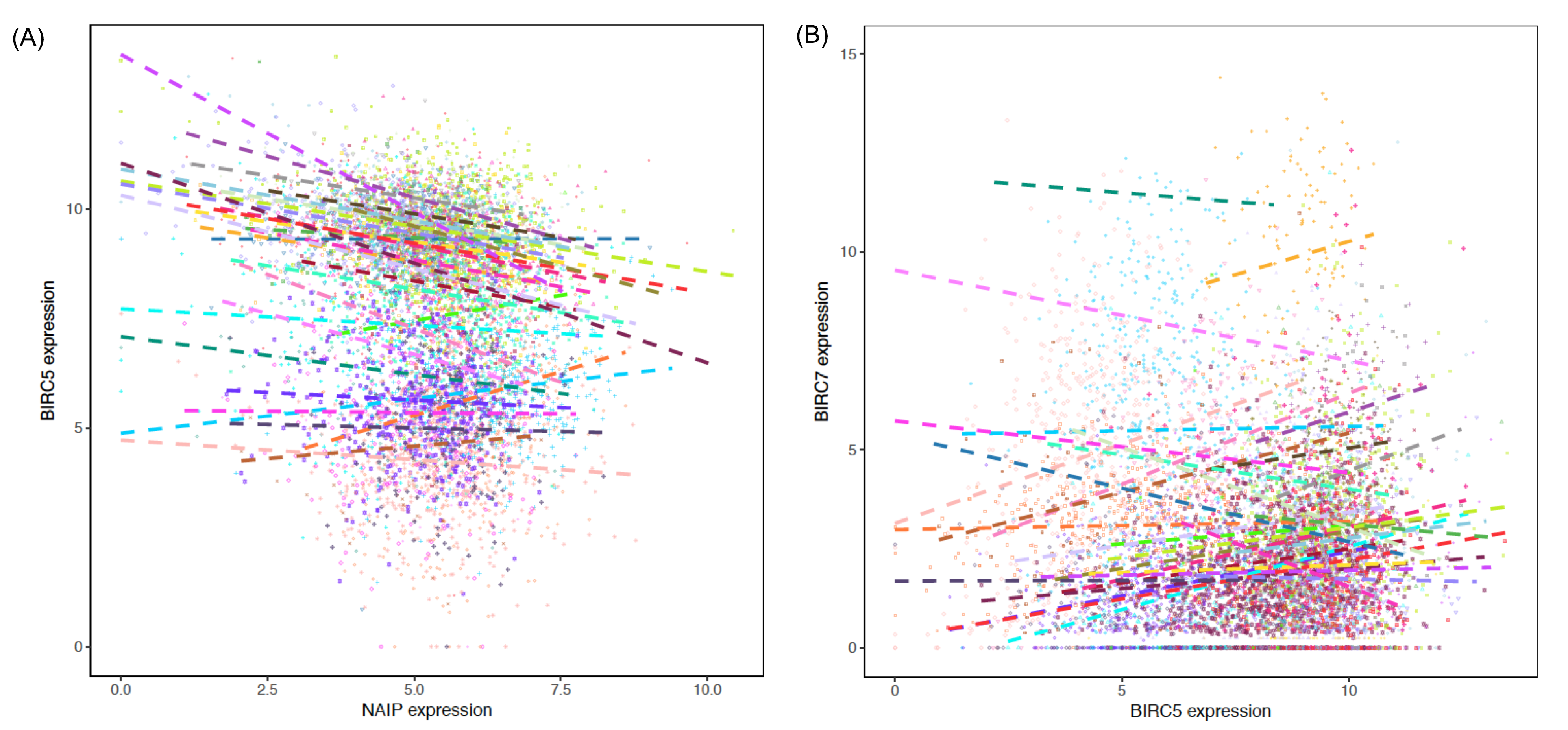

Supplement: Supplementary file 2 — Additional file 2: Figure S2. Example Co-expression Between IAPs. BIRC5 mostly showed anti-correlation with NAIP expression across different cancers (A). Between BIRC5 and BIRC7, both positive and negative correlations were observed across the 32 cancers (B). [file 12920_2020_661_MOESM2_ESM.png]

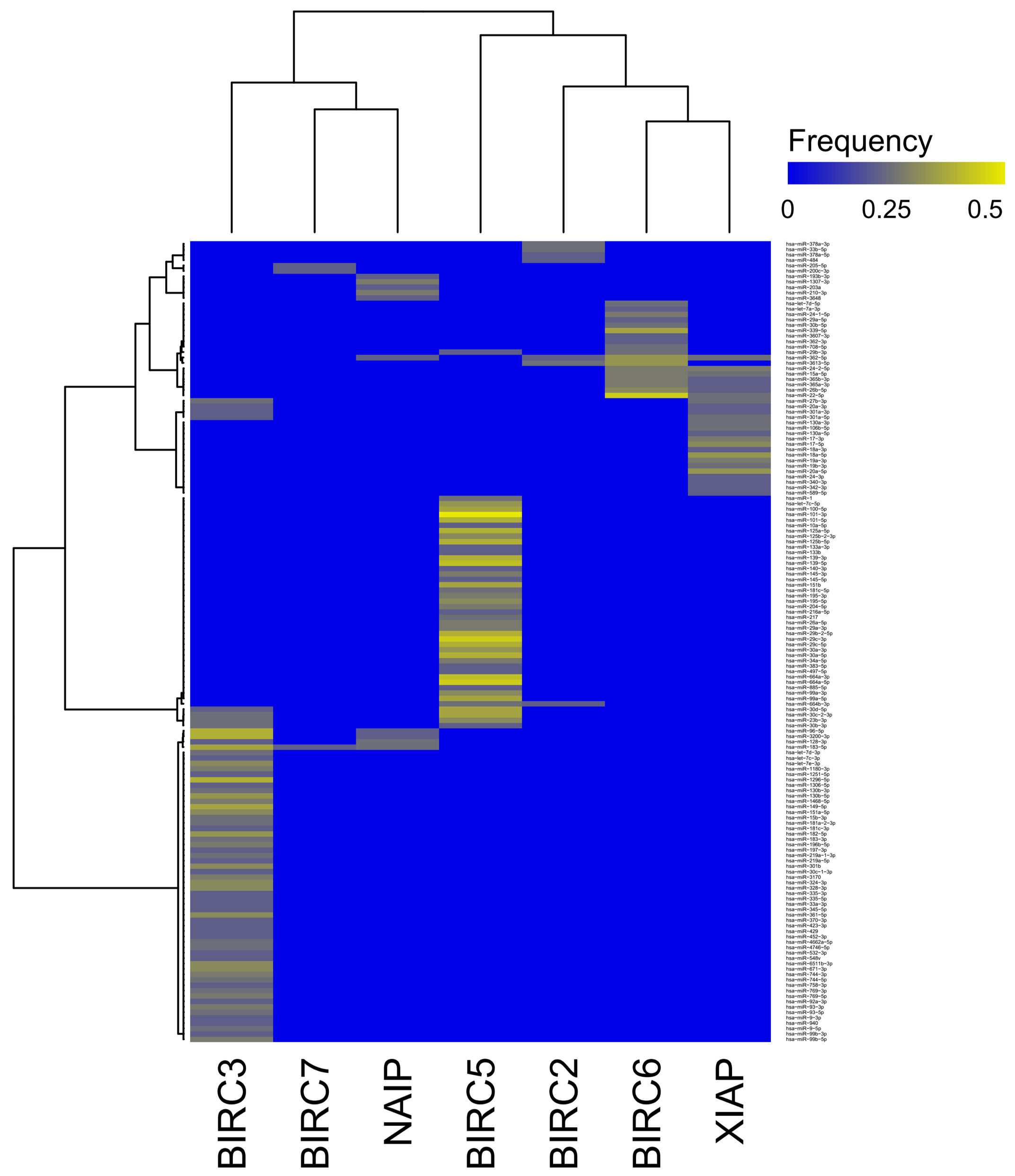

Supplement: Supplementary file 3 — Additional file 3: Figure S3. Regulation of IAPs by miRNAs. For each miRNA (rows) in a given IAP gene (columns), we compute frequency among 32 cancers that have significant anti-correlation between miRNA and IAP gene expression (correlation < − 0.2; adjusted p value < 0.05). [file 12920_2020_661_MOESM3_ESM.png]

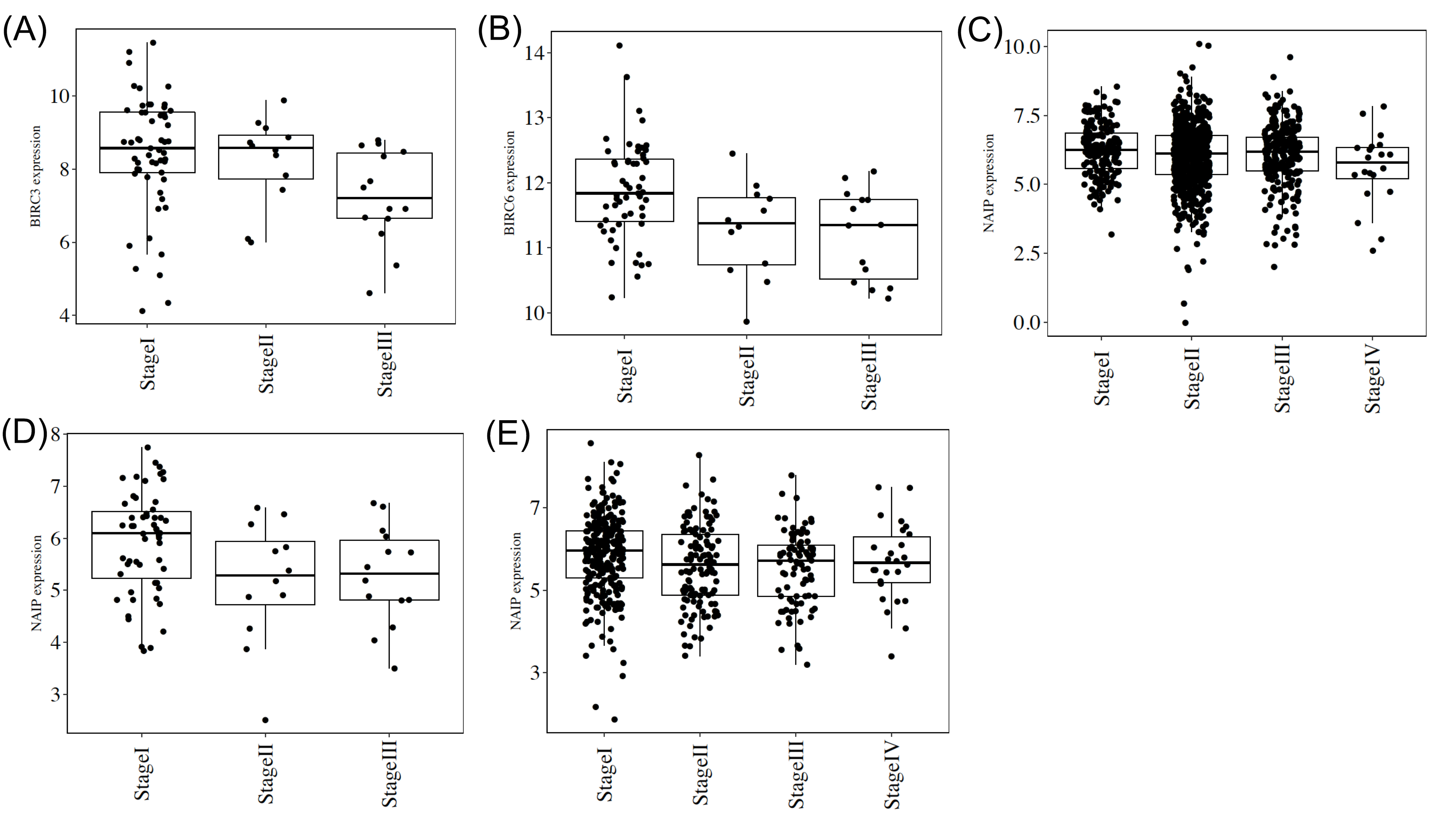

Supplement: Supplementary file 4 — Additional file 4: Figure S4. Example Negative Associations Between IAPs expression and Tumor Stage. (A), (B) and (C) were from TGCT, (D) was from BRCA and (E) was from LUAD. [file 12920_2020_661_MOESM4_ESM.png]

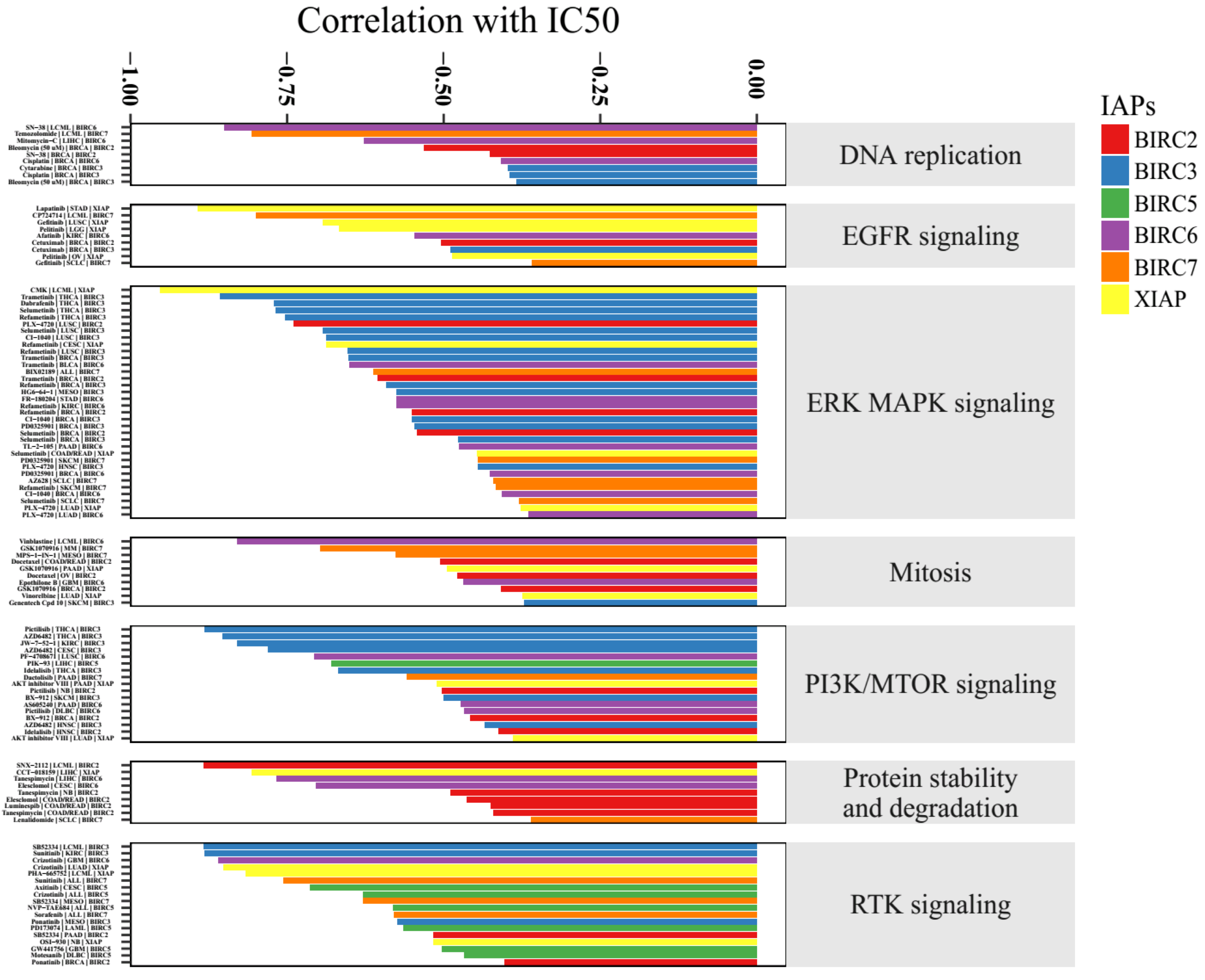

Supplement: Supplementary file 5 — Additional file 5: Figure S5. IAPs Determine Sensitivity to Other Inhibitors. [file 12920_2020_661_MOESM5_ESM.png]
